# Seizure-Induced Regulations of Amyloid-*β*, STEP_61_, and STEP_61_ Substrates Involved in Hippocampal Synaptic Plasticity

**DOI:** 10.1155/2016/2123748

**Published:** 2016-04-05

**Authors:** Sung-Soo Jang, Sara E. Royston, Gunhee Lee, Shuwei Wang, Hee Jung Chung

**Affiliations:** ^1^Department of Molecular and Integrative Physiology, University of Illinois at Urbana-Champaign, Urbana, IL 61801, USA; ^2^Neuroscience Program, University of Illinois at Urbana-Champaign, Urbana, IL 61801, USA; ^3^Medical Scholars Program, University of Illinois at Urbana-Champaign, Urbana, IL 61801, USA

## Abstract

Alzheimer's disease (AD) is a neurodegenerative disorder characterized by progressive cognitive decline. Pathologic accumulation of soluble amyloid-*β* (A*β*) oligomers impairs synaptic plasticity and causes epileptic seizures, both of which contribute to cognitive dysfunction in AD. However, whether seizures could regulate A*β*-induced synaptic weakening remains unclear. Here we show that a single episode of electroconvulsive seizures (ECS) increased protein expression of membrane-associated STriatal-Enriched protein tyrosine Phosphatase (STEP_61_) and decreased tyrosine-phosphorylation of its substrates N-methyl D-aspartate receptor (NMDAR) subunit GluN2B and extracellular signal regulated kinase 1/2 (ERK1/2) in the rat hippocampus at 2 days following a single ECS. Interestingly, a significant decrease in ERK1/2 expression and an increase in APP and A*β* levels were observed at 3-4 days following a single ECS when STEP_61_ level returned to the baseline. Given that pathologic levels of A*β* increase STEP_61_ activity and STEP_61_-mediated dephosphorylation of GluN2B and ERK1/2 leads to NMDAR internalization and ERK1/2 inactivation, we propose that upregulation of STEP_61_ and downregulation of GluN2B and ERK1/2 phosphorylation mediate compensatory weakening of synaptic strength in response to acute enhancement of hippocampal network activity, whereas delayed decrease in ERK1/2 expression and increase in APP and A*β* expression may contribute to the maintenance of this synaptic weakening.

## 1. Introduction

Alzheimer's disease (AD) is a neurodegenerative disorder characterized by progressive and irreversible cognitive decline [1]. Although AD pathology shows amyloid plaques that consist of insoluble amyloid-*β* (A*β*) [2], the abnormal accumulation of soluble A*β* oligomeric peptides correlates closely with cognitive dysfunction in early AD and disrupts synaptic plasticity [3], which is widely believed to underlie learning and memory [3, 4]. Specifically, soluble A*β* oligomers at pathologic levels inhibit long-term potentiation (LTP) and enhance long-term depression (LTD) of excitatory synaptic strength in the hippocampus, a brain region susceptible for neurodegeneration in AD [3]. Interestingly, the pathological accumulation of amyloid precursor protein (APP) and oligomeric A*β* also causes aberrant neuronal hyperexcitability in cortical and hippocampal neuronal networks of AD mouse models [5–9], consistent with the fact that humans and animal models with early-onset autosomal dominant familial AD have epileptic seizures [10–21]. Experimental inhibition of epileptic seizures prevents memory loss in AD transgenic model mice [17], suggesting that A*β*-induced aberrant increases in neuronal network activity also contribute to cognitive dysfunction in AD. However, whether seizures could regulate A*β*-induced synaptic weakening remains unclear.

STriatal-Enriched protein tyrosine Phosphatase 61 (STEP_61_) has recently emerged as a key regulator of A*β*-induced synaptic weakening [11, 22–26] and as a postsynaptic density protein highly regulated by hyperexcitability in hippocampal neurons [27]. Application of A*β* oligomers to cortical cultures reduces surface expression of glutamate-gated ionotropic receptors including N-methyl D-aspartate receptors (NMDARs) and *α*-amino-3-hydroxy-5-methyl-4-isoxazolepropionic acid receptors (AMPARs) by upregulating STEP_61_ activity [11, 22, 24–26]. While STEP_61_ levels are elevated in the cortices of several AD mouse models [22, 28], genetic ablation of STEP_61_ blocks A*β*-induced reduction in surface AMPAR and NMDAR expression [22, 25] and prevents memory loss and LTP impairment in AD mouse models [25, 26], suggesting a critical role of STEP_61_ in mediating A*β*-induced synaptic weakening and cognitive dysfunction in AD. We have also reported that prolonged enhancement of hippocampal network activity in primary culture leads to elevated STEP_61_ expression and STEP_61_-dependent reduction in tyrosine- (Tyr-) phosphorylation of its substrates, NMDAR subunit GluN2B and AMPAR subunit GluA2 [27].

Given that STEP_61_ level is elevated in human AD which displays epileptic seizures as a comorbid condition [10–13], we hypothesize that hippocampal neuronal hyperexcitability induced by seizures will increase STEP_61_ level, leading to compensatory downregulation of synaptic strength by dephosphorylating GluN2B, GluA2, and ERK1/2, key proteins critical for synaptic plasticity. To test this hypothesis, we induced a single electroconvulsive seizure (ECS) or chronic ECS (a single ECS each day for 7 consecutive days) in adult rats to elevate hippocampal network activity* in vivo* [29–32] and examined protein expression of STEP_61_ and A*β*, as well as tyrosine-phosphorylation of STEP_61_ substrates GluN2B, GluA2, and ERK1/2. ECS is an animal model for electroconvulsive therapy (ECT), which provides an efficient and relatively fast acting treatment for depression, anxiety, and other psychiatric conditions in humans [33]. During ECS, sufficient current administration reliably elicits nonrecurring stage 4-5 tonic-clonic seizures [29–32]. We chose ECS to globally elevate brain activity* in vivo* because a single ECS does not induce cell death or notable structural remodeling [33] which are evident in pilocarpine- or kainate-induced chronic epilepsy models [34, 35]. Furthermore, ECS does not involve invasive surgical methodologies as often used in kindling following intracranial electrode placement [34, 35].

We discovered that a single ECS increases the expression of membrane-associated STEP_61_ and decreases Tyr^1472^-phosphorylation of GluN2B and Tyr^204/187^-phosphorylation of ERK1/2 in the hippocampus at 48 hours (h) following a single ECS. Interestingly, upregulation of APP and A*β* levels was observed at 72–96 h following a single ECS when STEP_61_ level returned to the baseline. Chronic ECS results in a transient increase in APP and A*β* expression at 48 h and A*β* expression at 96 h following chronic ECS but did not alter STEP_61_ expression and Tyr-phosphorylation of its substrates. Furthermore, a persistent decrease in GluN2B expression was observed over a course of 96 h following chronic ECS. These results suggest that elevated expression of APP, A*β*, and STEP_61_ and dephosphorylation of GluN2B and ERK1/2 may contribute to compensatory weakening of synaptic strength in response to seizure-induced hippocampal network hyperexcitability.

## 2. Material and Methods

### 2.1. Materials

Antibodies used include anti-STEP_61_ (catalogue SC-23892, Santa Cruz), anti-GluN2B (#14544, Cell Signaling), anti-ERK1/2 (SC-154, Santa Cruz), anti-GluA2 (#5306, Cell Signaling), anti-APP (#SC-28365, Santa Cruz), and anti-*β*-actin (#4967, Cell Signaling). Phosphorylation site specific antibodies used include anti-GluN2B-pTyr^1472^ which recognizes phosphorylated Tyr-1472 of GluN2B (P1516-1472, PhosphoSolutions), anti-ERK1/2-pThr^202^/Tyr^204^ which recognizes phosphorylated Thr^202^/Tyr^204^ of ERK1 and Thr^185^/Tyr^187^ of ERK2 (#9106, Cell Signaling), anti-GluA2-p3Y which recognizes phosphorylated Tyr^869^, Tyr^873^, and Tyr^876^ (3Y) of GluA2 (#3921S, Cell Signaling), and anti-GluA2-pY^876^ which recognizes phosphorylated Tyr^876^ of GluA2 (#4027S, Cell Signaling).

### 2.2. Animals

The Institutional Animal Care and Use Committee at the University of Illinois at Urbana-Champaign approved all experimental procedures involving animals in this study.

### 2.3. Electroconvulsive Seizure (ECS)

Male Sprague-Dawley rats (bred in house; strain origin: Charles River Laboratories) were weaned at postnatal day (P) 28, housed in groups of 2–4 male littermates, and weighed 3 times per week. All animals were maintained in standard conditions with a 12-hour (h) light-dark cycle and ad libitum access to food and water. Male rats were used to eliminate potential confounding sex differences. Rats received either a single ECS or chronic ECS (a single ECS each day for 7 consecutive days) as previously described [30, 36, 37] with the following modification. All ECS were induced between 7:30 and 10:00 a.m. in adult rats weighing 200–250 grams. One at a time, rats were connected via ear-clip electrodes to a pulse generator (Ugo Basile, Comerio, Italy), and a 0.5 sec, 100 pulses/sec, 55 mA shock was delivered to elicit a stage 4-5 seizure. All ECS lasted <10 sec, after which rats were returned to their home cage. Sham “no seizure” animals (NS) were handled identically, including ear-clip electrodes attachment, but no current was delivered. One experiment for a single ECS or chronic ECS included one NS rat and one ECS-treated rat per each time point following the last ECS.

### 2.4. Whole Brain Lysate Preparation

At specific time point following a single ECS or chronic ECS, animals were sacrificed by CO_2_ inhalation and rapidly decapitated. The hippocampi were dissected from their brains and homogenized in ice-cold homogenization buffer (solution A) containing (in mM) 320 sucrose, 1 NaHCO_3_, 1 MgCl_2_, 0.5 CaCl_2_, 1 NaVO_3_, 10 Na_4_O_7_P_2_, 50 NaF, and Halt protease inhibitors (Thermo Fisher Scientific) (1.25 mL total volume per pair of hippocampi). The crude membrane fraction (P2) was isolated from the hippocampi homogenates as previously described [38] with the following modification. After centrifuging for 10 min at 1,400 g, the postnuclear supernatants were separated (S1) from insoluble tissue and nuclear pellet (P1). The pellets were reconstituted in ice-cold solution A (1.25 mL total volume per pair of hippocampi) and centrifuged for additional 10 min at 710 g. The resultant supernatant was combined with the S1 fraction, and the entire volume was then spun at 13,800 g for 10 min. The supernatant (S2) was removed, and the remaining pellet (P2 membrane fraction) was resuspended in ice-cold solution B containing (in mM) 320 sucrose, 1 NaHCO, 1 NaVO_3_, 10 Na_4_O_7_P_2_, 50 NaF, and protease inhibitor cocktails (1 mL total volume per pair of hippocampi). BCA assay (Pierce) analysis was performed to determine protein concentrations across samples, which were subsequently normalized to 1 mg/mL in solution B. The S1, S2, and P2 lysates were stored at −80°C until use.

### 2.5. Western Blot Analysis

After adding SDS sample buffer, the lysates (S1, P2, and S2) were heated at 37°C or 75°C for 30 min. Lysate samples were run on SDS-polyacrylamide gel electrophoresis (SDS-PAGE) gels and transferred to polyvinyl difluoride (PVDF) membrane (Millipore). Each gel contained lysates from one experiment for a single ECS or chronic ECS, including from one NS rat and one ECS-treated rat per each time point following the last ECS. Immunoblot analysis was performed as previously described [39, 40] with the following modifications. Each blot was blocked in 5% milk and 0.1% Tween-20 in Tris buffered saline (TBS) for 1 h and then incubated in primary antibodies in washing buffer (1% milk and 0.1% Tween-20 in TBS) overnight at 4°C. Primary antibodies used include anti-STEP_61_ (1 : 200), anti-GluN2B (1 : 1000), anti-GluA2 (1 : 1000), anti-APP (1 : 200), anti-*β*-actin (1 : 1000), anti-GluN2B-pTyr^1472^ (1 : 1000), anti-GluA2-p3Y (1 : 1000), anti-GluA2-pY^876^ (1 : 500), and anti-ERK1/2- pThr^202^/Tyr^204^ (1 : 1000). After incubating in HRP-conjugated secondary antibody in washing buffer for 1 h, blots were visualized with enhanced chemifluorescence substrate (ECL, Thermo Fisher Scientific) and developed with a Konica SRX-101A film processor. Densitometric quantification was performed with ImageJ Software (National Institutes of Health) as previously described [39, 40]. The band intensity of a protein of interest was divided by the *β*-actin band intensity per each time point. The ratio of NS control group was taken as 100%, and the ratio of ECS-treated group at each time point was normalized to the ratio of NS control to obtain the % of relative protein expression.

### 2.6. Statistical Analysis

**Figure 1:**
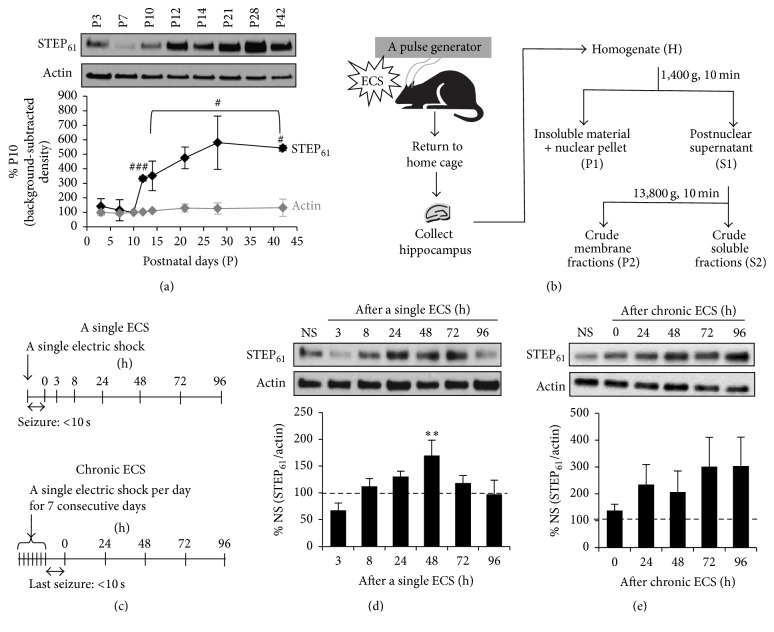
A single ECS but not chronic ECS transiently increases membrane-associated STEP_61_ expression in the hippocampus. (a) Hippocampal expression of STEP_61_ during postnatal development. Immunoblot analysis and quantification of STEP_61_ and *β*-actin from the crude membrane (P2) fractions of rat hippocampi were obtained at postnatal days (P) 3, 7, 10, 12, and 14 (*n* = 3 rats per time point) and 21, 28, and 42 (*n* = 2 rats per time point). Background-subtracted western blot band intensities of STEP_61_ and *β*-actin were normalized to those of P10 group, which was taken as 100%. STEP_61_ expression significantly increased from P10 to P12 by 3-fold (^###^ *p* < 0.005, *t*-test) and to P42 by 5-fold (^#^ *p* < 0.05, *t*-test). (b) Schematic workflow of an experiment from ECS induction in rats to biochemical fractionation of their hippocampi. (c) Schematic experimental design of a single ECS and chronic ECS (a single ECS per day for 7 consecutive days). (d-e) Immunoblot analysis of STEP_61_ in the hippocampal crude membrane (P2) fraction following a single ECS ((d) *n* = 5 rats per time point) and chronic ECS ((e) *n* = 6 rats per time point). Time points shown represent the duration after the induction of a single ECS (d) or chronic ECS (e) prior to brain removal. The ratio of the STEP_61_ band intensity over the *β*-actin band intensity was calculated per each time point and normalized to the ratio of “no seizure” (NS) sham group, which was taken as 100%. Data shown represent the mean band intensity ± SEM. (c) A single ECS transiently increases STEP_61_ expression in the hippocampus (^*∗∗*^ *p* < 0.01). (d) Chronic ECS does not significantly alter STEP_61_ expression in the hippocampus.

All data shown represent the mean value ± SEM. The number of rats is expressed as sample size *n*. Statistical analyses were performed with either Microsoft Excel or Origin (version 8.5; OriginLab). For most data sets, a priori value (^*∗*^ *p*) < 0.05 was considered statistically significant following one-way ANOVA and* post hoc* ANOVA tests (Fisher's test). For Figure 1(a), Student's *t*-test was used due to low sample size (*n* = 2-3 rats per postnatal day). For the statistical analysis of the levels of Tyr^204/187^-phosphorylated ERK1/2 in Figure 4(a) and A*β* in Figure 5(a), Student's *t*-test was used because one-way ANOVA and* post hoc* ANOVA tests (Fisher's test) were not adequate to perform in the data sets that contained a large variability when 5 sets of independent experiments for a single ECS were compared. For Student's *t*-test, a priori value (^#^ *p*) < 0.05 was considered statistically significant.

## 3. Results and Discussion

### 3.1. A Single ECS but Not Chronic ECS Transiently Increased STEP_61_ Expression in the Hippocampus

To test whether elevation of hippocampal network activity* in vivo* regulates STEP_61_ level, ECS was induced in rats and their hippocampal membrane fractions were collected for western blot analysis. First, we examined the developmental expression of STEP_61_ in male rats (Figure 1(a)). Hippocampal STEP_61_ expression began to increase at P12 compared to P3–P10 (332.8 ± 16.3% of P10, *p* < 0.005, *t*-test compared to P10) (Figure 1(a)). Although highly variable, STEP_61_ expression steadily increased from P12 to P28 and reached a plateau at P42 with statistical significance (543.5 ± 18.0% of P10, *p* < 0.05, *t*-test compared to P10 and P12) (Figure 1(a)). Since hippocampal STEP_61_ expression stabilized by P42 (Figure 1(a)), ECS were induced only in male rats that were at >P42 and weighed 220–240 g. For the induction of ECS, male rats received a single electric shock (0.5 sec, 100 pulses/sec, 55 mA) for once (a single ECS) or 7 consecutive days (chronic ECS) as previously described [30, 36] (Figures 1(b) and 1(c)). “No seizure” animals (NS) were handled identically, but no current was delivered. STEP_61_ protein level in the crude membrane P2 fractions of hippocampus progressively increased up to 169.9 ± 28.3% by 48 h following induction of a single ECS compared to NS groups (*p* < 0.01, Figure 1(d); see Supplemental Figure  1 of the Supplementary Material available online at http://dx.doi.org/10.1155/2016/2123748). Interestingly, elevated STEP_61_ level decreased back to the level of NS group by 72 h after a single ECS (Figure 1(d), Supplemental Figure  1). Although an increasing trend has been observed for STEP_61_ expression over the course of 96 h following chronic ECS, this trend did not reach statistical significance due to a large standard deviation (Figure 1(e), Supplemental Figure  1). A single ECS or chronic ECS did not alter STEP_61_ expression in postnuclear supernatant (S1) fraction (Supplemental Figure  2). Taken together, these data indicate that a single ECS but not chronic ECS caused a transient but significant increase in membrane-associated STEP_61_ expression in the hippocampus* in vivo*.

### 3.2. A Single ECS but Not Chronic ECS Transiently Decreased Tyr^1472^-Phosphorylation of GluN2B in the Hippocampus

**Figure 2:**
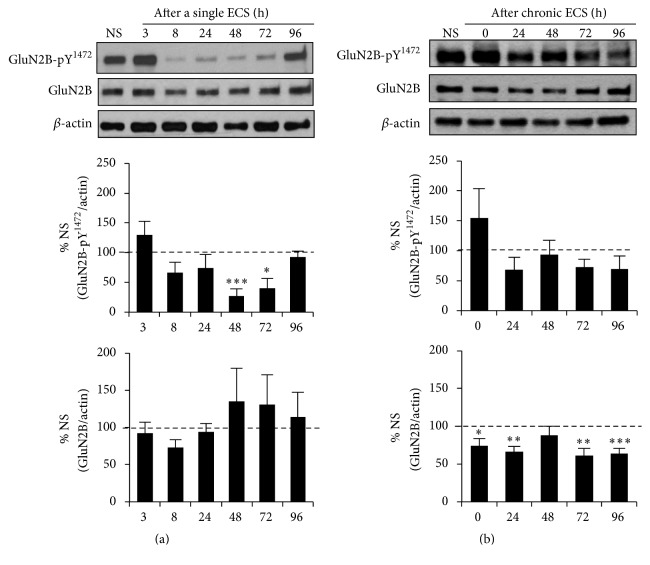
A single ECS but not chronic ECS transiently decreases the level of Tyr^1472^-phosphorylated GluN2B in the hippocampus. Immunoblot analysis for the phosphorylation of GluN2B at Tyr^1472^ (Y^1472^) and total GluN2B expression in the hippocampal crude membrane (P2) fraction following a single ECS (*n* = 5 rats per time point) (a) and chronic ECS (*n* = 6 rats per time point) (b). The ratio of the Tyr^1472^-phosphorylated GluN2B band intensity over the *β*-actin band intensity (top graphs) and the ratio of total GluN2B band intensity over the *β*-actin band intensity (bottom graphs) were calculated per each time point and normalized to that of “no seizure” (NS) sham group. Data shown represent the mean band intensity ± SEM. (a) A single ECS transiently decreases the level of Tyr^1472^-phosphorylated GluN2B in the hippocampus at 48 h (^*∗∗∗*^ *p* < 0.005) and 72 h (^*∗*^ *p* < 0.05) following a single ECS. (b) Chronic ECS significantly decreases total GluN2B expression over the time course of 96 h in the hippocampus (^*∗*^ *p* < 0.05, ^*∗∗*^ *p* < 0.01, and ^*∗∗∗*^ *p* < 0.005).

Enriched in the postsynaptic density, STEP_61_ dephosphorylates NMDAR subunit GluN2B at Tyr^1472^, leading to internalization of GluN2B-containing NMDAR [11, 22, 24, 25, 41, 42]. We hypothesized that a single ECS-induced increase in STEP_61_ expression would decrease Tyr^1472^-phosphorylation of GluN2B in the hippocampus. Consistent with our hypothesis, western blot analysis of hippocampal P2 lysates revealed a significant reduction in the level of Tyr^1472^-phosphorylated GluN2B (GluN2B-pY^1472^) compared to NS group from 48 to 72 h following a single ECS (Supplemental Figure  3), with the most reduction seen at 48 h (Figure 2(a), 27.0 ± 12.7% of NS, *p* < 0.005) when STEP_61_ expression was transiently enhanced (Figure 1(d)). The level of Tyr^1472^-phosphorylated GluN2B was returned to the level of NS group by 96 h after a single ECS (Figure 2(a), Supplemental Figure  3) when elevated STEP_61_ level decreased back to the level of NS group (Figure 1(d)). Total GluN2B expression did not change following a single ECS (Figure 2(a), Supplemental Figure  3). Although chronic ECS did not alter the level of Tyr^1472^-phosphorylated GluN2B compared to NS control (Figure 2(b), Supplemental Figure  3), there was a modest but significant reduction in total GluN2B expression from 0 h to 24 h and 72 h to 96 h following chronic ECS compared to NS control (Figure 2(b), 96 h: 63.8 ± 6.7%, *p* < 0.005). These data indicate that a single ECS transiently reduced Tyr^1472^-phosphorylation of GluN2B whereas chronic ECS persistently reduced total GluN2B expression in the hippocampus.

### 3.3. A Single ECS but Not Chronic ECS Increased the Level of Tyr^876^-Phosphorylated GluA2 in the Hippocampus

**Figure 3:**
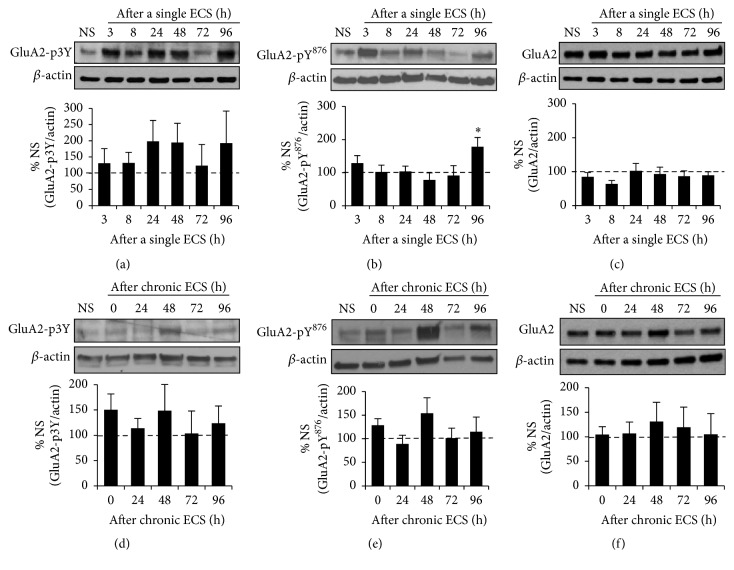
A single ECS but not chronic ECS increases the level of Tyr^876^-phosphorylated GluA2 in the hippocampus. Immunoblot analysis for phosphorylation of GluA2 at Tyr^876^ (Y^876^) or 3Tyr (3Y: Tyr^869^, Tyr^873^, and Tyr^876^) and total GluA2 expression in the hippocampal crude membrane (P2) fraction following a single ECS (*n* = 5 rats per time point) (a–c) and chronic ECS (*n* = 5 rats for 72 h time point and *n* = 6 rats per all other time points) (d–f). The ratio of the phosphorylated GluA2 band intensity over the *β*-actin band intensity (a-b, d-e) and the ratio of total GluA2 band intensity over the *β*-actin band intensity (c, f) were calculated per each time point and normalized to that of “no seizure” (NS) sham group. Data shown represent the mean band intensity ± SEM. (a–c) A single ECS increases the level of Tyr^876^-phosphorylated GluA2 at 96 h time point ((b) ^*∗*^ *p* < 0.05) but does not alter the level of 3Tyr-phosphorylated GluA2 and total GluA2. (d–f) Chronic ECS does not change Tyr-phosphorylation of GluA2 (d-e) and total GluA2 expression (f).

STEP_61_ reduces Tyr-phosphorylation of AMPAR subunit GluA2 and mediates AMPAR internalization upon acute stimulation of group 1 metabotropic glutamate receptors (mGluR) and application of A*β* [24, 26]. Although it is unclear which specific Tyr residue (s) in GluA2 is directly dephosphorylated by STEP_61_, AMPAR internalization is reported to involve dephosphorylation of Tyr^869^, Tyr^873^, and Tyr^876^ (3Tyr) within the intracellular GluA2 C-terminal region (GluA-p3Y) [43]. We therefore hypothesized that a single ECS-induced increase in STEP_61_ expression would decrease the level of 3Tyr-phosphorylated GluA2 as well as Tyr^876^-phosphorylated GluA2 in the hippocampus. There was an increasing trend for the level of 3Tyr-phosphorylated GluA2 over the course of 96 h following a single ECS compared to NS control, although this increase did not reach statistical significance due to a large standard deviation (Figure 3(a), Supplemental Figure  4). To our surprise, the level of Tyr^876^-phosphorylated GluA2 was unaltered at 48 h following a single ECS (Figure 3(b), Supplemental Figure  4, 77.8 ± 21.4% of NS, *p* > 0.05) when STEP_61_ expression was significantly increased compared to NS control (Figure 1(d)). Instead, the level of Tyr^876^-phosphorylated GluA2 was significantly increased by 2-fold at 96 h following a single ECS (Figure 3(b), 178.6 ± 27.5% of NS, *p* < 0.05) when STEP_61_ expression was similar to that of NS control (Figure 1(d)). A single ECS had no effect on total GluA2 expression (Figure 3(c), Supplemental Figure  4). Chronic ECS did not alter the levels of 3Tyr-phosphorylated GluA2, Tyr^876^-phosphorylated GluA2, and total GluA2 (Figures 3(d)–3(f), Supplemental Figure  5). These data indicate that a single ECS regulates Tyr^876^-phosphorylation of GluA2 in the hippocampus.

### 3.4. A Single ECS and Chronic ECS Differently Altered Tyr^204/187^-Phosphorylation of ERK1/2 in the Hippocampus

**Figure 4:**
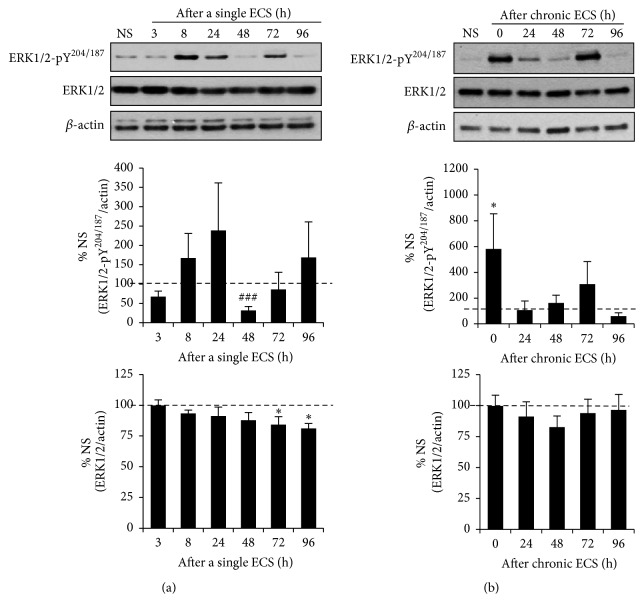
A single ECS and chronic ECS differently alter Tyr^204/187^-phosphorylation of ERK1/2 in the hippocampus. Immunoblot analysis for the phosphorylation of ERK1/2 at Tyr^204/187^ (Y^204/187^) and total ERK1/2 expression in the hippocampal crude membrane (P2) fraction following a single ECS (*n* = 5 rats per time point) (a) and chronic ECS (*n* = 6 rats per time point) (b). The ratio of the Tyr^204/187^-phosphorylated ERK1/2 band intensity over the *β*-actin band intensity (top graphs) and the ratio of total ERK1/2 band intensity over the *β*-actin band intensity (bottom graphs) were calculated per each time point and normalized to that of “no seizure” (NS) sham group. Data shown represent the mean band intensity ± SEM. (a) A single ECS transiently decreases the level of Tyr^204/187^-phosphorylated ERK1/2 in the hippocampus at 48 h (^###^ *p* < 0.005, *t*-test) and total ERK1/2 expression at 72–96 h (^*∗*^ *p* < 0.05) following a single ECS. (b) Chronic ECS significantly increases the level of Tyr^204/187^-phosphorylated ERK1/2 at 0 h following chronic ECS (^*∗*^ *p* < 0.05) but has no effect on total GluN2B expression in the hippocampus.

STEP_61_-mediated dephosphorylation of ERK1/2 at Tyr^204/187^ inactivates ERK1/2, opposing synaptic strengthening during LTP [44, 45]. Thus, we next tested whether a single ECS-induced increase in STEP_61_ expression would decrease Tyr^204/187^-phosphorylation of ERK1/2 in the hippocampus. There was an initial increasing trend for the level of Tyr^204/187^-phosphorylated ERK1/2 (ERK-pY^204/187^) until 24 h following a single ECS (Figure 4(a), *p* > 0.05). At 48 h following a single ECS when STEP_61_ expression was significantly increased (Figure 1(d)), the level of Tyr^204/187^-phosphorylated ERK1/2 was markedly reduced to 31.9 ± 10.2% of NS (*p* < 0.005, Figure 4(a), Supplemental Figure  6). As STEP_61_ level reduced to those of NS groups from 48 h to 96 h after a single ECS (Figure 1(d)), the level of Tyr^204/187^-phosphorylated ERK1/2 also gradually increased to the level of NS groups (Figure 4(a), Supplemental Figure  6). Total ERK1/2 expression was significantly reduced at 72 h to 96 h following a single ECS (Figure 4(a), Supplemental Figure  6; 72 h: 84.2 ± 6.4% of NS, *p* < 0.05, 96 h: 81.1 ± 4.0% of NS, *p* < 0.05). Interestingly, chronic ECS caused about a 6-fold increase in the level of Tyr^204/187^-phosphorylated ERK1/2 at 0 h time point compared to NS group (Figure 4(b), Supplemental Figure  6; 0 h: 580.5 ± 273.6% of NS, *p* < 0.05). However, this initial increase was decreased to the level of NS group by 24 h after chronic ECS (Figure 4(b), *p* < 0.05 between 0 h and 24 h). There was no change in total ERK1/2 expression in the hippocampus following chronic ECS (Figure 4(b), Supplemental Figure  6). Collectively, these results show that a single ECS and chronic ECS dynamically modulate Tyr^204/187^-phosphorylation of ERK1/2.

### 3.5. A Single ECS and Chronic ECS Increased the Expression of APP and A*β* Oligomers in the Hippocampus

**Figure 5:**
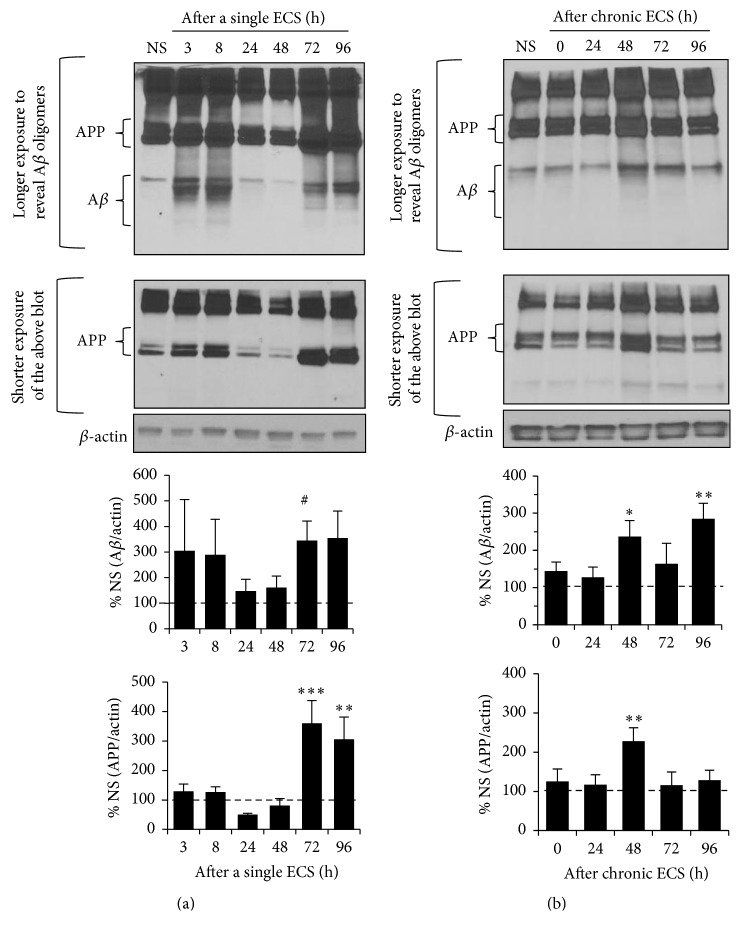
A single ECS and chronic ECS increase APP and oligomeric A*β* expression in the hippocampus. Immunoblot analysis of APP and oligomeric A*β* in the hippocampal crude soluble (S2) fraction following a single ECS (*n* = 5 rats per time point) (a) and chronic ECS (*n* = 6 rats per time point) (b). The ratio of the A*β* band intensity over the *β*-actin band intensity (top graphs) and the ratio of the APP band intensity over the *β*-actin band intensity (bottom graphs) were calculated per each time point and normalized to that of “no seizure” (NS) sham group. Data shown represent the mean band intensity ± SEM. (a) A single ECS increases A*β* expression at 72 h (^#^ *p* < 0.05, *t*-test) and APP expression at 72–96 h following a single ECS (^*∗∗*^ *p* < 0.01, ^*∗∗∗*^ *p* < 0.005). (b) Chronic ECS increases A*β* expression at 48 h and 96 h, as well as APP expression at 48 h following chronic ECS (^*∗*^ *p* < 0.05, ^*∗∗*^ *p* < 0.01).

The A*β* peptide is derived from the cleavage of the amyloid precursor protein (APP) by *β*-secretase and *γ*-secretase at the Golgi and to a lesser extent endoplasmic reticulum [46]. A*β* oligomers have been shown to reduce surface expression of NMDARs and AMPARs by upregulating STEP_61_ activity [11, 22, 24–26]. Furthermore, an A*β*-mediated disruption of the proteasome leads to increased STEP_61_ levels in human AD brains and AD mouse models [11, 22, 25, 26]. Since A*β* is produced and secreted from neurons in response to synaptic activity [47–49], we next examined whether ECS could increase the production of A*β* peptides by performing western blotting in crude soluble S2 fractions of the hippocampus. Upon induction of a single ECS, the level of A*β* oligomers increased by 3-fold compared to the NS group at 72 h (Figure 5(a), Supplemental Figure  7; A*β*-72 h: 345.3 ± 75.8%, *p* < 0.05) when STEP_61_ level is similar to that of NS group (Figure 1(d)). APP expression was also increased by 3-fold at 72–96 h following a single ECS (Figure 5(a), Supplemental Figure  7; APP-72 h: 406.5 ± 83.0%, *p* < 0.005, APP-96 h: 363.6 ± 81.9%, *p* < 0.01). Chronic ECS also caused a 2- to 3-fold increase in the expression of A*β* oligomers at 48 h and 96 h (Figure 5(b), Supplemental Figure  8; A*β*-48 h: 235.4 ± 44.7%, *p* < 0.05, A*β*-96 h: 283.5 ± 43.1%, *p* < 0.01) and a 2-fold increase in the expression of APP at 48 h following chronic ECS (Figure 5(b), Supplemental Figure  8; 228.0 ± 34.3%  *p* < 0.005). These data indicate that both a single ECS and a chronic ECS led to a delayed increase in the levels of APP and A*β* oligomers in the hippocampus.

### 3.6. The Physiologic Consequences of STEP_61_ Regulation in the Hippocampus by ECS

**Figure 6:**
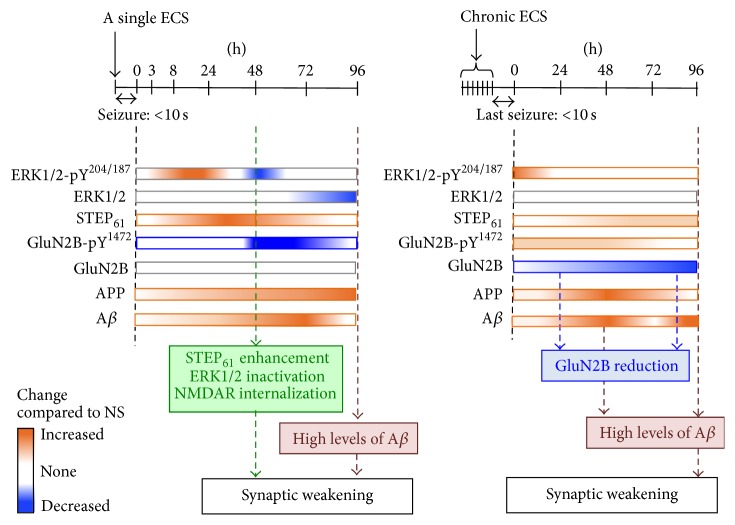
Model by which seizure-induced changes in A*β*, STEP_61_, and Tyr-phosphorylation of STEP_61_ lead to synaptic weakening in the hippocampus. A single ECS increases STEP_61_ expression and decreases Tyr-phosphorylation of NMDAR subunit GluN2B and ERK1/2 in the hippocampus at 48 h time point, leading to synaptic weakening via NMDAR internalization and ERK1/2 inactivation. A delayed decrease in ERK1/2 expression as well as a delayed enhancement of APP and A*β* expression at 72–96 h following a single ECS maintains this synaptic weakening. Chronic ECS-induced increase in APP expression and A*β* production as well as persistent decrease in total GluN2B level leads to synaptic weakening.

We show that STEP_61_ level was markedly increased in rat hippocampus at 48 h after a single induction of ECS (Figure 1(d)), which induces global elevation of hippocampal neuronal activity [33]. Consistent with this increase in STEP_61_ expression, the level of Tyr^1472^-phosphorylated GluN2B was reduced at 48–72 h following a single ECS without altering total GluN2B expression (Figure 2(a)). Considering that STEP_61_-mediated dephosphorylation of GluN2B leads to internalization of GluN2B-containing NMDARs [11], upregulation of STEP_61_ (Figure 1(d)) may serve as a compensatory mechanism to reduce surface density of NMDARs in the hippocampus in response to seizures (Figure 6). Consistent with the previous report on ECS-induced decreases in PSD-95 and GluN2A/B expression [50], chronic ECS caused a persistent decrease in total GluN2B expression over the course of 96 h following chronic ECS (Figure 2(b)). NMDAR activation requires coincident binding of glutamate and membrane depolarization produced by opening of AMPARs [51]. Hence, although chronic ECS did not alter GluA2 level (Figure 3(f)), a persistent decline in GluN2B expression (Figure 2(b)) could facilitate synaptic weakening in response to repetitive seizures.

STEP_61_ mediates AMPAR internalization upon mGluR activation and A*β* application by dephosphorylating GluA2 [24, 26], suggesting a possibility that a single ECS-induced increase in STEP_61_ expression could lead to synaptic weakening by decreasing Tyr-phosphorylation of GluA2. Unexpectedly, the level of Tyr^876^-phosphorylated GluA2 was enhanced at 96 h following a single ECS (Figure 3(b)). No significant changes were seen in the level of 3Tyr-phosphorylated GluA2 and total GluA2 following a single ECS (Figures 3(a) and 3(c)). Since the level of Tyr^876^-phosphorylated GluA2 was unaltered at 48 h following a single ECS when STEP_61_ was increased (Figures 1(d) and 3(b)), Tyr^876^ of GluA2 might not have been directly regulated by STEP_61_. It is also possible that STEP_61_ dephosphorylates a specific residue within the 3Tyr motif, but that kinase-mediated phosphorylation of another residue within the same motif could mask the STEP_61_ effect. Identification of specific phosphorylation sites regulated by STEP_61_ may aid future studies to dissect the role of STEP_61_ in ECS-induced regulation of GluA2 Tyr-phosphorylation.

The level of Tyr^204/187^-phosphorylated ERK1/2 was markedly decreased at 48 h following a single ECS (Figure 4(a)), when STEP_61_ expression was at its peak (Figure 1(d)). Since STEP_61_-mediated dephosphorylation of ERK1/2 at Tyr^204/187^ inactivates ERK1/2 [44, 45], our results suggest a significant reduction in ERK1/2 activity by ECS-induced upregulation of STEP_61_. The total ERK1/2 expression was also reduced at 72–96 h following a single ECS (Figure 4(a)). Considering that activation of ERK1/2 drives synaptic delivery of AMPAR [52] and activity-dependent regulation of gene transcription during LTP [53], ERK1/2 inactivation at 48 h and ERK1/2 reduction at 72–96 h following a single ECS would also facilitate synaptic weakening (Figure 6). Interestingly, chronic ECS caused a 6-fold increase in the level of Tyr^204/187^-phosphorylated ERK1/2 at 0 h following chronic ECS compared to NS control, which was returned to the level of NS control by 24 h after chronic ECS (Figure 4(b)). These temporal changes in ERK1/2 activity are consistent with previous reports in cultured neurons that ERK1/2 undergoes rapid activation in response to glutamate stimulation, followed by a STEP_61_-dependent delayed inactivation to baseline [44, 54, 55]. Taken together, our results suggest that upregulation of STEP_61_ and downregulation of its substrates critical for synaptic plasticity may provide efficient means to mediate synaptic weakening (Figure 6).

### 3.7. The Physiologic Consequences of APP and A*β* Regulation in the Hippocampus by ECS

Previous studies have shown that application of A*β* oligomers activates STEP_61_, which subsequently leads to internalization of NMDAR and AMPAR [11, 22]. We speculate that the delayed 3-fold increase in APP and A*β* expression at 72–96 h following a single ECS (Figure 5(a)) would enhance STEP_61_ activity, leading to a persistent reduction in NMDAR and AMPAR surface expression at these time points when STEP_61_ levels returned back to NS control levels (Figure 6). Such persistent decrease in synaptic strength is expected to lead to the elimination of synapses [56–58]. Indeed, decreases in synapse density are evident in the hippocampi of patients with early AD and correlate strongly with memory impairment [59–61]. Furthermore, a single ECS has been shown to cause memory deficits in rats when it was administered right after the hippocampus-dependent learning experience [50, 62], consistent with clinical observations of retrograde amnesia as one severe side effect for electroconvulsive therapy in humans [63]. Hence, it will be interesting to test if seizure-induced increase in APP and A*β* expression and downregulation of NMDAR and ERK1/2 through STEP_61_ could be the basis of cognitive deficits in early AD and ECT.

Interestingly, a transient 2-fold increase in APP and A*β* expression was observed at 48 h after chronic ECS compared to NS control, which was followed by a 3-fold increase in A*β* expression at 96 h time point (Figure 5(b)). The initial increase in APP expression could be the basis for the delayed increase in A*β* levels. Animals administered with chronic ECS display increased dentate granule cell neurogenesis [64] and molecular layer mossy fiber sprouting [65, 66]. Since APP regulates neurite outgrowth as well as cell adhesion and promotes neuronal survival [67–70], chronic ECS-induced increase in APP expression may regulate hippocampal neurogenesis and mossy fiber sprouting. Interestingly, similar 3-4-fold increase in APP levels has been found in the postmortem temporal lobe from patients with early AD [71] and from patients with intractable temporal lobe epilepsy with abnormal neurite outgrowth [72]. The AD transgenic mouse models with elevated APP expression display spontaneous seizures, sharp wave discharges, and mossy fiber sprouting as well as ectopic expression of inhibitory neuropeptides in their hippocampus [5, 14, 15, 17–21]. Importantly, hippocampal neurons in transgenic APP-overexpressing AD mice display hyperexcitability well before plaque formation [6]. Since A*β* application increases the activity of excitatory neurons in acute brain slices [20] and neuronal activity stimulates synthesis and synaptic release of A*β* [47–49], we speculate that chronic ECS-induced initial increase in APP expression may result in neuronal hyperexcitability, which in turn causes heightened A*β* expression, ultimately leading to pathologic positive feedback loop of A*β* production [73].

### 3.8. The Mechanisms Underlying ECS-Induced Expression of STEP_61_ and APP

Previous studies have shown that STEP_61_ expression is regulated by multiple mechanisms. STEP_61_ is locally translated in dendrites upon mGluR5 activation through a mechanism dependent on ERK1/2 phosphorylation [24]. Interestingly, fragile X mental retardation protein (FMRP) binds to and inhibits translation of STEP mRNA [74] whereas genetic ablation of FMRP leads to increased STEP expression [75, 76]. Hence, it is possible that the initial increasing trend in ERK1/2 phosphorylation induced by a single ECS (Figure 4(a)) could increase STEP_61_ expression by 48 h (Figure 1(d)) by triggering local dendritic synthesis of STEP_61_ upon FMRP inhibition. In addition, STEP_61_ undergoes proteasome-dependent degradation upon polyubiquitination [77], suggesting another possibility that a single ECS could elevate STEP_61_ expression by inhibiting proteasomal STEP_61_ degradation. Lastly, both a single ECS and a chronic ECS stimulate robust induction of immediate early genes and subsequent downstream genes important for neural plasticity [30, 31, 66, 78–83]. Considering that 48 h blockade of neuronal activity or NMDAR in cultured hippocampal neurons leads to a significant reduction in STEP_61_ mRNA and protein expression [27], a single ECS may stimulate transcription of STEP_61_ in the hippocampus through NMDAR activation. Further investigation is needed to investigate if enhancement of hippocampal network activity upon a single ECS increases STEP_61_ protein level by enhancing transcription and translation of STEP_61_ and/or inhibiting its proteasomal degradation.

It is unclear how a single and a chronic ECS caused a delayed increase in APP and A*β* expression in the hippocampus (Figure 5). Previous studies have shown that APP synthesis and processing are stimulated by interleukin-1 (IL-1) [84–86], which is synthesized and released from activated microglia [87, 88]. Consistently, neuronal expression of APP is associated with heightened IL-1 immunoreactivity in human temporal lobe epilepsy [72]. Interestingly, activated microglia are found in the hippocampus 24 h after a single or repeated ECS, and the number of activated microglial cells remained increased for weeks after ECS [89]. Though highly speculative, it is possible that ECS-induced persistent activation of microglia could stimulate IL-1 synthesis and release, leading to delayed APP production in neurons following a single ECS or a chronic ECS.

Chronic ECS did not induce significant alterations in the levels of STEP_61_, GluA2, and ERK1/2 compared to NS groups (Figures 1–4). While chronic ECS is therapeutically used to reduce stress [33], it is also possible that the “no seizure” (NS) animals might have been hyperstressed by the repeated exposures to handling and the ECS apparatus, even though current was not delivered. Considering the interdependence of stress and STEP_61_ expression [90], heightened stress in the NS animals in combination with dampened stress levels in chronic ECS-received rats may account for the lack of effects on STEP_61_ regulation following chronic ECS administration.

## 4. Conclusion

Here, we show that a single ECS transiently increases protein expression of membrane-associated STEP_61_ and decreases Tyr-phosphorylation of NMDAR subunit GluN2B and ERK1/2 in the hippocampus. A delayed decrease in the levels of ERK1/2 as well as a delayed enhancement of APP and A*β* expression is also seen in the hippocampus following a single ECS. Chronic ECS treatment also leads to a persistent decrease in GluN2B level and a transient increase in APP and A*β* production. To our knowledge this is the first study reporting the temporal expression of APP, A*β*, STEP_61_ and its substrates at various time points following a single ECS and chronic ECS. We propose that this regulation causes a transient weakening of synaptic strength to combat global enhancement of hippocampal neuronal activity induced by ECS. This regulation may also contribute to hippocampus-dependent memory loss induced by ECS, supporting antiepileptic drugs as potential therapy for cognitive dysfunction in early AD [17]. Given that A*β*-induced increase in STEP_61_ expression is involved in NMDAR and AMPAR internalization during synaptic weakening in AD [11, 22, 24–26], the work reported here emphasizes the need to dissect the detailed molecular mechanisms underlying activity-dependent regulation of STEP_61_. These mechanistic insights may help to explain the heightened STEP_61_ expression present in AD [11, 22, 25] and fragile X syndrome [76] which have epileptic seizures as comorbid conditions.

## Supplementary Material

The Supplementary Material shows all raw data collected for Figures 1-5 in the hippocampal membrane P2 fractions of male rats treated with a single ECS and chronic ECS.

## References

[1] McKhann G., Drachman D., Folstein M., Katzman R., Price D., Stadlan E. M. (1984). Clinical diagnosis of Alzheimer's disease: report of the NINCDS-ADRDA Work Group under the auspices of Department of Health and Human Services Task Force on Alzheimer's Disease. *Neurology*.

[2] Kidd M. (1964). Alzheimer's disease—an electron microscopical study. *Brain*.

[3] Hsieh H., Boehm J., Sato C. (2006). AMPAR removal underlies A*β*-induced synaptic depression and dendritic spine loss. *Neuron*.

[4] Lynch M. A. (2004). Long-term potentiation and memory. *Physiological Reviews*.

[5] Palop J. J., Mucke L. (2010). Amyloid-beta-induced neuronal dysfunction in Alzheimer's disease: from synapses toward neural networks. *Nature Neuroscience*.

[6] Busche M. A., Chen X., Henning H. A. (2012). Critical role of soluble amyloid-*β* for early hippocampal hyperactivity in a mouse model of Alzheimer's disease. *Proceedings of the National Academy of Sciences of the United States of America*.

[7] Lalonde R., Fukuchi K.-I., Strazielle C. (2012). Neurologic and motor dysfunctions in APP transgenic mice. *Reviews in the Neurosciences*.

[8] Jankowsky J. L., Slunt H. H., Gonzales V. (2005). Persistent amyloidosis following suppression of A*β* production in a transgenic model of Alzheimer disease. *PLoS Medicine*.

[9] Vogt D. L., Thomas D., Galvan V., Bredesen D. E., Lamb B. T., Pimplikar S. W. (2011). Abnormal neuronal networks and seizure susceptibility in mice overexpressing the APP intracellular domain. *Neurobiology of Aging*.

[10] Amatniek J. C., Hauser W. A., DelCastillo-Castaneda C. (2006). Incidence and predictors of seizures in patients with Alzheimer's disease. *Epilepsia*.

[11] Snyder E. M., Nong Y., Almeida C. G. (2005). Regulation of NMDA receptor trafficking by amyloid-*β*. *Nature Neuroscience*.

[12] Larner A. J., Doran M. (2006). Reply to Dr Raux *et al*.: molecular diagnosis of autosomal dominant early onset Alzheimer's disease: an update (*J Med Genet* 2005;42:793–5). *Journal of Medical Genetics*.

[13] Jayadev S., Leverenz J. B., Steinbart E. (2010). Alzheimer's disease phenotypes and genotypes associated with mutations in presenilin 2. *Brain*.

[14] Westmark C. J., Westmark P. R., Beard A. M., Hildebrandt S. M., Malter J. S. (2008). Seizure susceptibility and mortality in mice that over-express amyloid precursor protein. *International Journal of Clinical and Experimental Pathology*.

[15] Lalonde R., Dumont M., Staufenbiel M., Strazielle C. (2005). Neurobehavioral characterization of APP23 transgenic mice with the SHIRPA primary screen. *Behavioural Brain Research*.

[16] Palop J. J., Chin J., Roberson E. D. (2007). Aberrant excitatory neuronal activity and compensatory remodeling of inhibitory hippocampal circuits in mouse models of Alzheimer's disease. *Neuron*.

[17] Sanchez P. E., Zhu L., Verret L. (2012). Levetiracetam suppresses neuronal network dysfunction and reverses synaptic and cognitive deficits in an Alzheimer's disease model. *Proceedings of the National Academy of Sciences of the United States of America*.

[18] Verret L., Mann E. O., Hang G. B. (2012). Inhibitory interneuron deficit links altered network activity and cognitive dysfunction in Alzheimer model. *Cell*.

[19] Corbett B. F., Leiser S. C., Ling H.-P. (2013). Sodium channel cleavage is associated with aberrant neuronal activity and cognitive deficits in a mouse model of Alzheimer's disease. *The Journal of Neuroscience*.

[20] Minkeviciene R., Rheims S., Dobszay M. B. (2009). Amyloid *β*-induced neuronal hyperexcitability triggers progressive epilepsy. *Journal of Neuroscience*.

[21] Ziyatdinova S., Gurevicius K., Kutchiashvili N. (2011). Spontaneous epileptiform discharges in a mouse model of Alzheimer's disease are suppressed by antiepileptic drugs that block sodium channels. *Epilepsy Research*.

[22] Kurup P., Zhang Y., Xu J. (2010). A*β*-mediated NMDA receptor endocytosis in Alzheimer's disease involves ubiquitination of the tyrosine phosphatase STEP61. *Journal of Neuroscience*.

[23] Kurup P., Zhang Y., Venkitaramani D. V., Xu J., Lombroso P. J. (2010). The role of STEP in Alzheimer's disease. *Channels*.

[24] Zhang Y., Venkitaramani D. V., Gladding C. M. (2008). The tyrosine phosphatase STEP mediates AMPA receptor endocytosis after metabotropic glutamate receptor stimulation. *Journal of Neuroscience*.

[25] Zhang Y., Kurup P., Xu J. (2010). Genetic reduction of striatal-enriched tyrosine phosphatase (STEP) reverses cognitive and cellular deficits in an Alzheimer’s disease mouse model. *Proceedings of the National Academy of Sciences of the United States of America*.

[26] Zhang Y., Kurup P., Xu J. (2011). Reduced levels of the tyrosine phosphatase STEP block beta amyloid-mediated GluA1/GluA2 receptor internalization. *Journal of Neurochemistry*.

[27] Jang S. S., Royston S. E., Xu J. (2015). Regulation of STEP61 and tyrosine-phosphorylation of NMDA and AMPA receptors during homeostatic synaptic plasticity. *Molecular Brain*.

[28] Chin J., Palop J. J., Puoliväli J. (2005). Fyn kinase induces synaptic and cognitive impairments in a transgenic mouse model of Alzheimer's disease. *Journal of Neuroscience*.

[29] Cardoso A., Carvalho L. S., Lukoyanova E. A., Lukoyanov N. V. (2009). Effects of repeated electroconvulsive shock seizures and pilocarpine-induced status epilepticus on emotional behavior in the rat. *Epilepsy and Behavior*.

[30] Madsen T. M., Newton S. S., Eaton M. E., Russell D. S., Duman R. S. (2003). Chronic electroconvulsive seizure up-regulates *β*-catenin expression in rat hippocampus: role in adult neurogenesis. *Biological Psychiatry*.

[31] Segi-Nishida E., Warner-Schmidt J. L., Duman R. S. (2008). Electroconvulsive seizure and VEGF increase the proliferation of neural stem-like cells in rat hippocampus. *Proceedings of the National Academy of Sciences of the United States of America*.

[32] Shibley H., Smith B. N. (2002). Pilocarpine-induced status epilepticus results in mossy fiber sprouting and spontaneous seizures in C57BL/6 and CD-1 mice. *Epilepsy Research*.

[33] Duman R. S., Vaidya V. A. (1998). Molecular and cellular actions of chronic electroconvulsive seizures. *The Journal of ECT*.

[34] Grone B. P., Baraban S. C. (2015). Animal models in epilepsy research: legacies and new directions. *Nature Neuroscience*.

[35] Kandratavicius L., Balista P. A., Lopes-Aguiar C. (2014). Animal models of epilepsy: use and limitations. *Neuropsychiatric Disease and Treatment*.

[36] Chen A. C.-H., Shin K.-H., Duman R. S., Sanacora G. (2001). ECS-induced mossy fiber sprouting and BDNF expression are attenuated by ketamine pretreatment. *The Journal of ECT*.

[37] Chen A. C., Eisch A. J., Sakai N., Takahashi M., Nestler E. J., Duman R. S. (2001). Regulation of GFRalpha-1 and GFRalpha-2 mRNAs in rat brain by electroconvulsive seizure. *Synapse*.

[38] Carlin R. K., Grab D. J., Cohen R. S., Siekevitz P. (1980). Isolation and characterization of postsynaptic densities from various brain regions: enrichment of different types of postsynaptic densities. *Journal of Cell Biology*.

[39] Lee K. Y., Chung H. J. (2014). NMDA receptors and L-type voltage-gated Ca^2+^ channels mediate the expression of bidirectional homeostatic intrinsic plasticity in cultured hippocampal neurons. *Neuroscience*.

[40] Lee K. Y., Royston S. E., Vest M. O. (2015). *N*-methyl-D-aspartate receptors mediate activity-dependent down-regulation of potassium channel genes during the expression of homeostatic intrinsic plasticity. *Molecular Brain*.

[41] Braithwaite S. P., Adkisson M., Leung J. (2006). Regulation of NMDA receptor trafficking and function by striatal-enriched tyrosine phosphatase (STEP). *European Journal of Neuroscience*.

[42] Venkitaramani D. V., Moura P. J., Picciotto M. R., Lombroso P. J. (2011). Striatal-enriched protein tyrosine phosphatase (STEP) knockout mice have enhanced hippocampal memory. *European Journal of Neuroscience*.

[43] Hayashi T., Huganir R. L. (2004). Tyrosine phosphorylation and regulation of the AMPA receptor by SRC family tyrosine kinases. *Journal of Neuroscience*.

[44] Paul S., Nairn A. C., Wang P., Lombroso P. J. (2003). NMDA-mediated activation of the tyrosine phosphatase STEP regulates the duration of ERK signaling. *Nature Neuroscience*.

[45] Venkitaramani D. V., Paul S., Zhang Y. (2009). Knockout of STriatal enriched protein tyrosine phosphatase in mice results in increased ERK1/2 phosphorylation. *Synapse*.

[46] Puzzo D., Arancio O. (2013). Amyloid-*β* peptide: Dr. Jekyll or Mr. Hyde?. *Journal of Alzheimer's Disease*.

[47] Kamenetz F., Tomita T., Hsieh H. (2003). APP processing and synaptic function. *Neuron*.

[48] Cirrito J. R., Yamada K. A., Finn M. B. (2005). Synaptic activity regulates interstitial fluid amyloid-*β* levels in vivo. *Neuron*.

[49] Bero A. W., Yan P., Roh J. H. (2011). Neuronal activity regulates the regional vulnerability to amyloid-*β* deposition. *Nature Neuroscience*.

[50] Yao Z., Guo Z., Yang C. (2010). Phenylbutyric acid prevents rats from electroconvulsion-induced memory deficit with alterations of memory-related proteins and tau hyperphosphorylation. *Neuroscience*.

[51] Paoletti P., Neyton J. (2007). NMDA receptor subunits: function and pharmacology. *Current Opinion in Pharmacology*.

[52] Patterson M. A., Szatmari E. M., Yasuda R. (2010). AMPA receptors are exocytosed in stimulated spines and adjacent dendrites in a Ras-ERK-dependent manner during long-term potentiation. *Proceedings of the National Academy of Sciences of the United States of America*.

[53] Wiegert J. S., Bading H. (2011). Activity-dependent calcium signaling and ERK-MAP kinases in neurons: a link to structural plasticity of the nucleus and gene transcription regulation. *Cell Calcium*.

[54] Paul S., Connor J. A. (2010). NR2B-NMDA receptor-mediated increases in intracellular Ca^2+^ concentration regulate the tyrosine phosphatase, STEP, and ERK MAP kinase signaling. *Journal of Neurochemistry*.

[55] Valjent E., Pascoli V., Svenningsson P. (2005). Regulation of a protein phosphatase cascade allows convergent dopamine and glutamate signals to activate ERK in the striatum. *Proceedings of the National Academy of Sciences of the United States of America*.

[56] Zhou Q., Homma K. J., Poo M.-M. (2004). Shrinkage of dendritic spines associated with long-term depression of hippocampal synapses. *Neuron*.

[57] Bastrikova N., Gardner G. A., Reece J. M., Jeromin A., Dudek S. M. (2008). Synapse elimination accompanies functional plasticity in hippocampal neurons. *Proceedings of the National Academy of Sciences of the United States of America*.

[58] Lamprecht R., LeDoux J. (2004). Structural plasticity and memory. *Nature Reviews Neuroscience*.

[59] Masliah E., Mallory M., Alford M. (2001). Altered expression of synaptic proteins occurs early during progression of Alzheimer's disease. *Neurology*.

[60] Scheff S. W., Price D. A., Schmitt F. A., Mufson E. J. (2006). Hippocampal synaptic loss in early Alzheimer's disease and mild cognitive impairment. *Neurobiology of Aging*.

[61] Scheff S. W., Price D. A., Schmitt F. A., Dekosky S. T., Mufson E. J. (2007). Synaptic alterations in CA1 in mild Alzheimer disease and mild cognitive impairment. *Neurology*.

[62] Misanin J. R., Miller R. R., Lewis D. J. (1968). Retrograde amnesia produced by electroconvulsive shock after reactivation of a consolidated memory trace. *Science*.

[63] Lisanby S. H. (2007). Electroconvulsive therapy for depression. *The New England Journal of Medicine*.

[64] Scott B. W., Wojtowicz J. M., Burnham W. M. (2000). Neurogenesis in the dentate gyrus of the rat following electroconvulsive shock seizures. *Experimental Neurology*.

[65] Gombos Z., Spiller A., Cottrell G. A., Racine R. J., McIntyre Burnham W. (1999). Mossy fiber sprouting induced by repeated electroconvulsive shock seizures. *Brain Research*.

[66] Vaidya V. A., Terwilliger R. Z., Duman R. S. (2000). Alterations in heavy and light neurofilament proteins in hippocampus following chronic ECS administration. *Synapse*.

[67] Whitson J. S., Selkoe D. J., Cotman C. W. (1989). Amyloid *β* protein enhances the survival of hippocampal neurons in vitro. *Science*.

[68] Araki W., Kitaguchi N., Tokushima Y. (1991). Trophic effect of *β*-amyloid precursor protein on cerebral cortical neurons in culture. *Biochemical and Biophysical Research Communications*.

[69] Milward E. A., Papadopoulos R., Fuller S. J. (1992). The amyloid protein precursor of Alzheimer's disease is a mediator of the effects of nerve growth factor on neurite outgrowth. *Neuron*.

[70] Schubert D. (1989). The biological roles of heparan sulfate proteoglycans in the nervous system. *Neurobiology of Aging*.

[71] Roberts G. W., Nash M., Ince P. G., Royston M. C., Gentleman S. M. (1993). On the origin of alzheimer’s disease a hypothesis. *NeuroReport*.

[72] Sheng J. G., Boop F. A., Mrak R. E., Griffin W. S. T. (1994). Increased neuronal *β*-amyloid precursor protein expression in human temporal lobe epilepsy: association with interleukin-1*α* immunoreactivity. *Journal of Neurochemistry*.

[73] Noebels J. (2011). A perfect storm: converging paths of epilepsy and Alzheimer's dementia intersect in the hippocampal formation. *Epilepsia*.

[74] Darnell J. C., Van Driesche S. J., Zhang C. (2011). FMRP stalls ribosomal translocation on mRNAs linked to synaptic function and autism. *Cell*.

[75] Goebel-Goody S. M., Baum M., Paspalas C. D. (2012). Therapeutic implications for striatal-enriched protein tyrosine phosphatase (STEP) in neuropsychiatric disorders. *Pharmacological Reviews*.

[76] Goebel-Goody S. M., Wilson-Wallis E. D., Royston S., Tagliatela S. M., Naegele J. R., Lombroso P. J. (2012). Genetic manipulation of STEP reverses behavioral abnormalities in a fragile X syndrome mouse model. *Genes, Brain and Behavior*.

[77] Xu J., Kurup P., Zhang Y. (2009). Extrasynaptic NMDA receptors couple preferentially to excitotoxicity via calpain-mediated cleavage of STEP. *The Journal of Neuroscience*.

[78] de Jong J. O. Z., Arts B., Boks M. P. (2014). Epigenetic effects of electroconvulsive seizures. *Journal of ECT*.

[79] Ma D. K., Jang M.-H., Guo J. U. (2009). Neuronal activity-induced Gadd45b promotes epigenetic DNA demethylation and adult neurogenesis. *Science*.

[80] Tsankova N. M., Kumar A., Nestler E. J. (2004). Histone modifications at gene promoter regions in rat hippocampus after acute and chronic electroconvulsive seizures. *Journal of Neuroscience*.

[81] Altar C. A., Laeng P., Jurata L. W. (2004). Electroconvulsive seizures regulate gene expression of distinct neurotrophic signaling pathways. *The Journal of Neuroscience*.

[82] Newton S. S., Collier E. F., Hunsberger J. (2003). Gene profile of electroconvulsive seizures: induction of neurotrophic and angiogenic factors. *The Journal of Neuroscience*.

[83] Zetterström T. S. C., Pei Q., Grahame-Smith D. G. (1998). Repeated electroconvulsive shock extends the duration of enhanced gene expression for BDNF in rat brain compared with a single administration. *Molecular Brain Research*.

[84] Goldgaber D., Harris H. W., Hla T. (1989). Interleukin 1 regulates synthesis of amyloid *β*-protein precursor mRNA in human endothelial cells. *Proceedings of the National Academy of Sciences of the United States of America*.

[85] Donnelly R. J., Friedhoff A. J., Beer B., Blume A. J., Vitek M. P. (1990). Interleukin-1 stimulates the beta-amyloid precursor protein promoter. *Cellular and Molecular Neurobiology*.

[86] Buxbaum J. D., Oishi M., Chen H. I. (1992). Cholinergic agonists and interleukin 1 regulate processing and secretion of the Alzheimer beta/A4 amyloid protein precursor. *Proceedings of the National Academy of Sciences of the United States of America*.

[87] Hetier E., Ayala J., Denefle P. (1988). Brain macrophages synthesize interleukin-1 and interleukin-1 mRNAs in vitro. *Journal of Neuroscience Research*.

[88] Righi M., Mori L., De Libero G. (1989). Monokine production by microglial cell clones. *European Journal of Immunology*.

[89] Jinno S., Kosaka T. (2008). Reduction of Iba1-expressing microglial process density in the hippocampus following electroconvulsive shock. *Experimental Neurology*.

[90] Yang C.-H., Huang C.-C., Hsu K.-S. (2012). A critical role for protein tyrosine phosphatase nonreceptor type 5 in determining individual susceptibility to develop stress-related cognitive and morphological changes. *The Journal of Neuroscience*.

