# Supplementary material for: Seizure-Induced Regulations of Amyloid-β, STEP61, and STEP61 Substrates Involved in Hippocampal Synaptic Plasticity
Source: Neural Plast. 2016 Apr 5;2016:2123748. doi: 10.1155/2016/2123748 (PMC4835651; doi:10.1155/2016/2123748)

Supplemental Figure 1

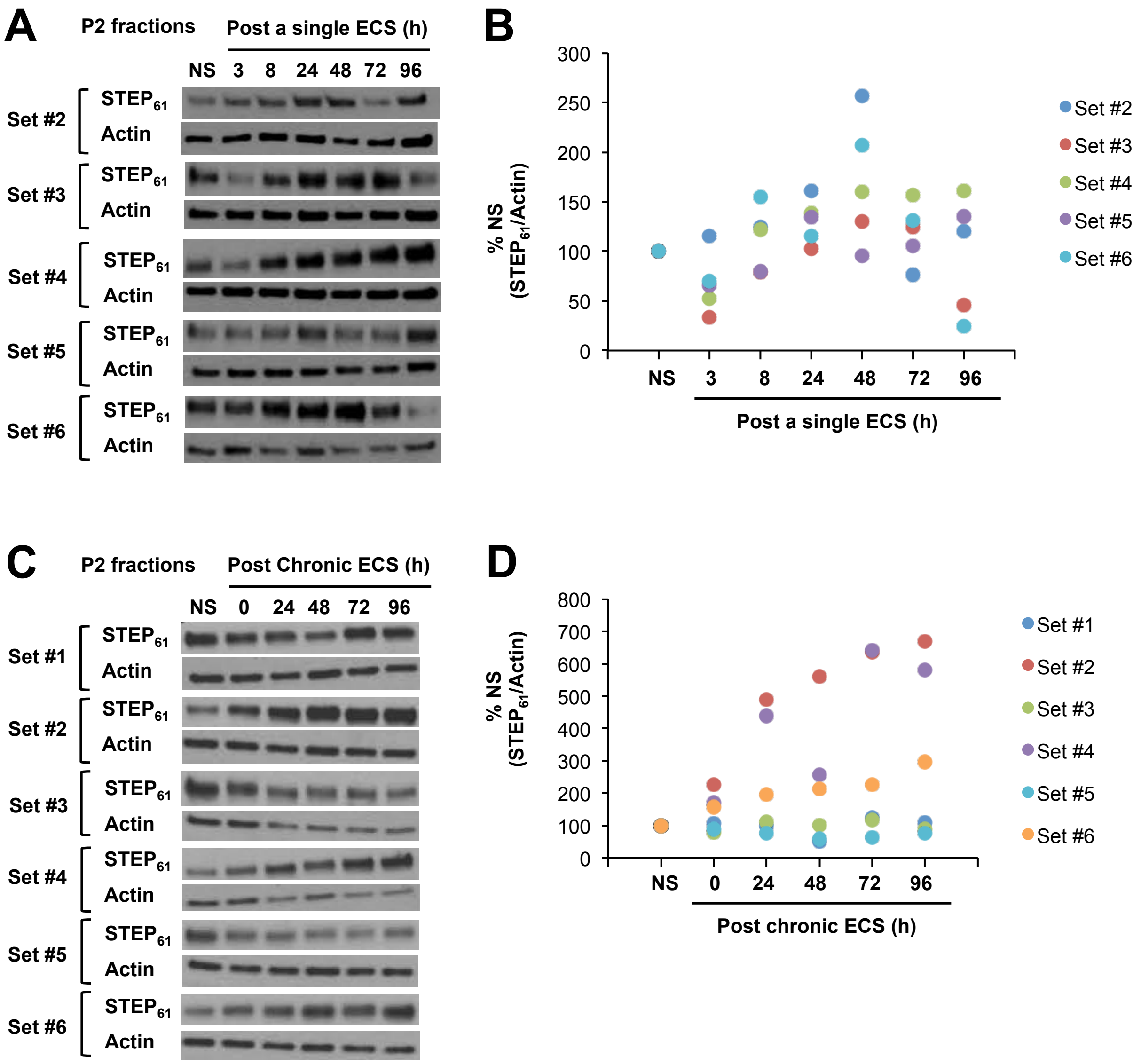

Supplemental Figure 2

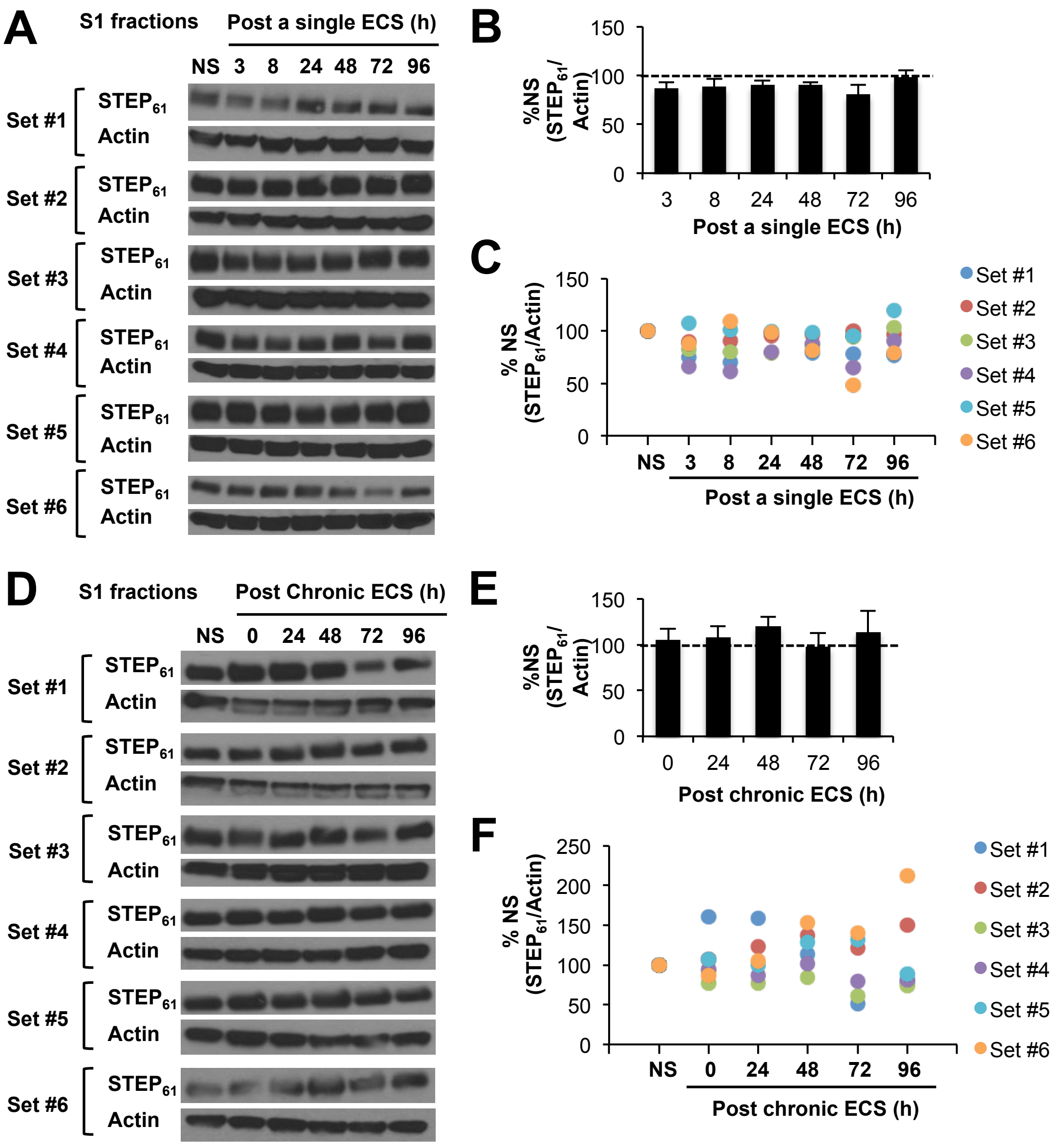

Supplemental Figure 3

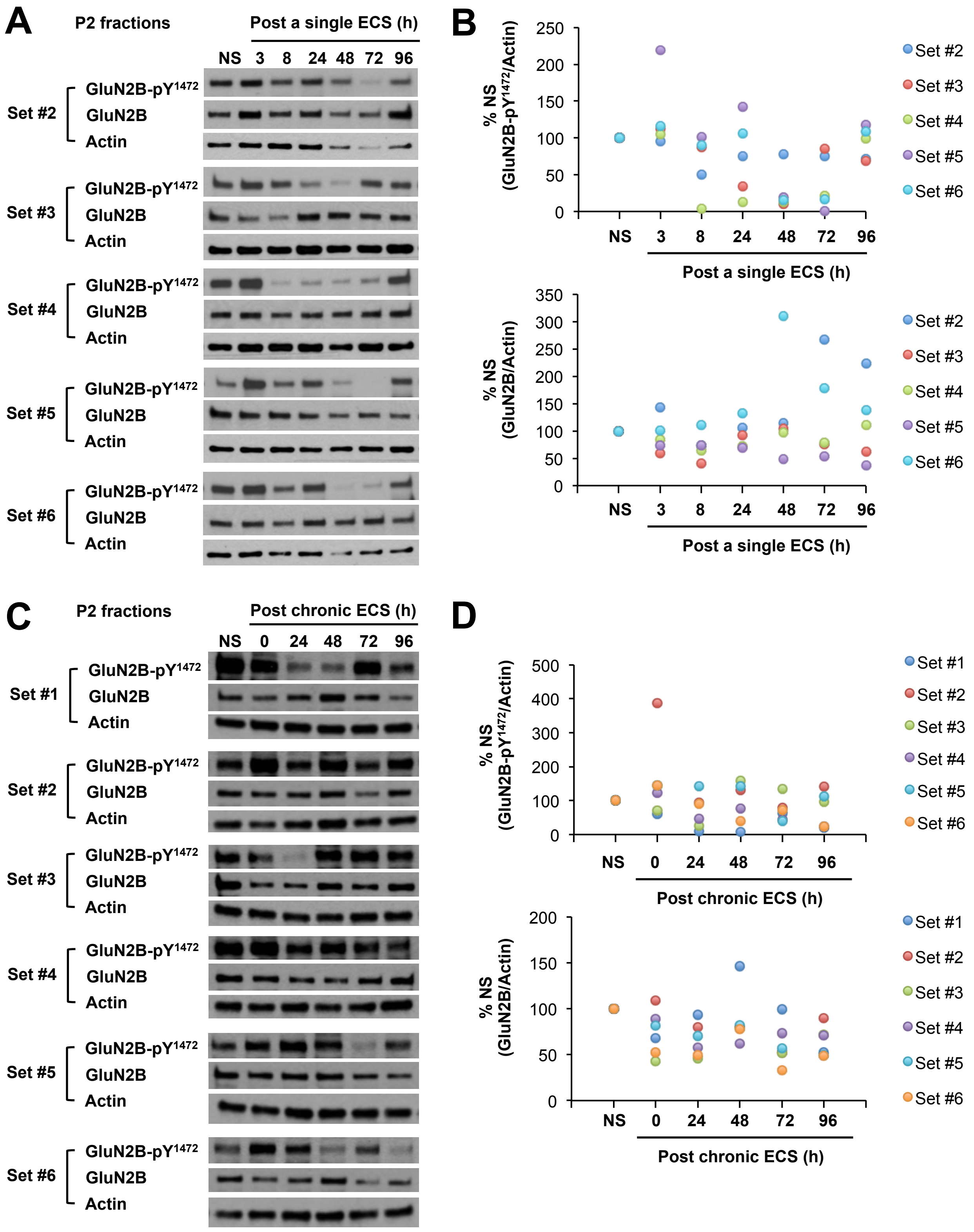

Supplemental Figure 4

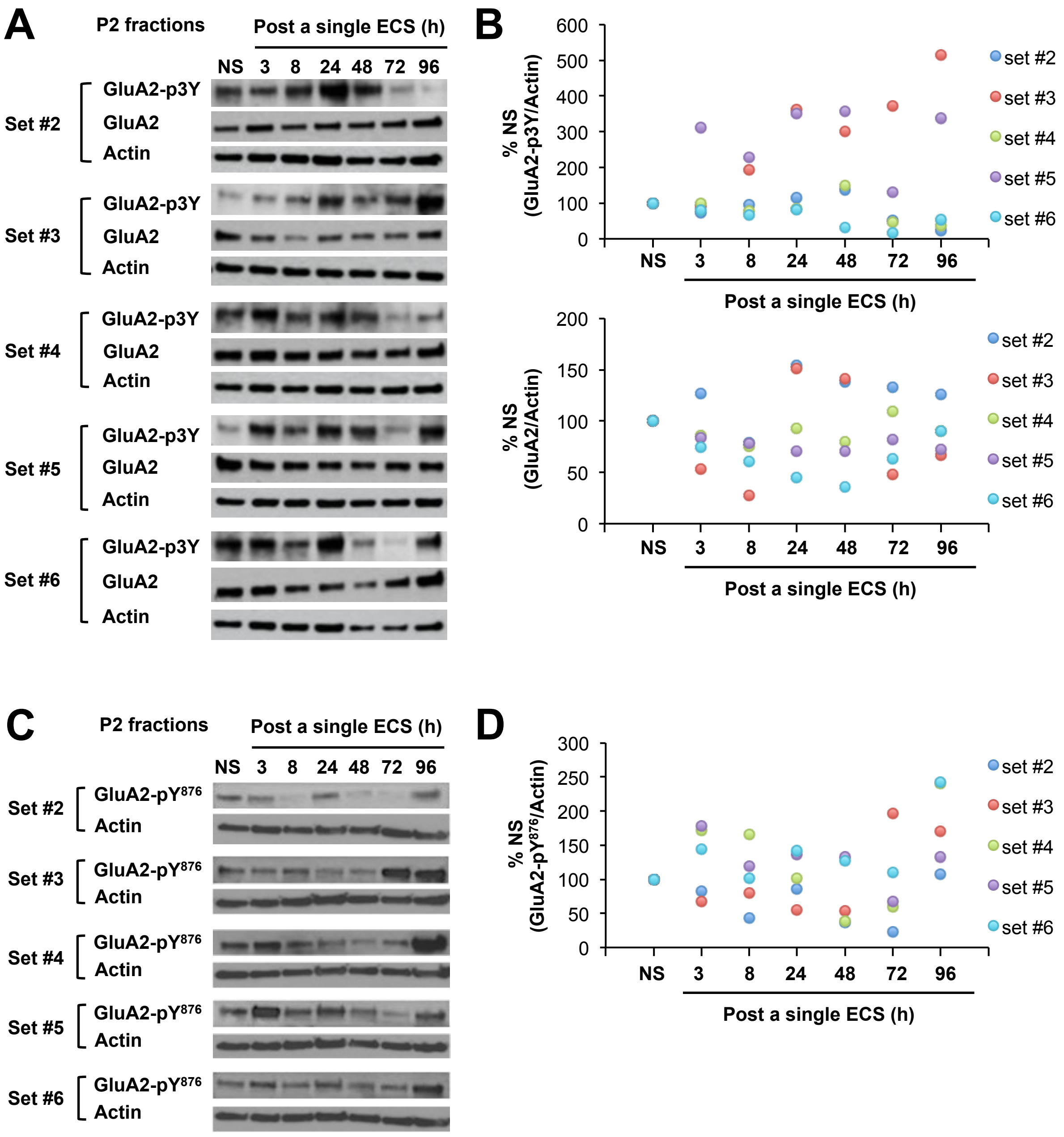

Supplemental Figure 5

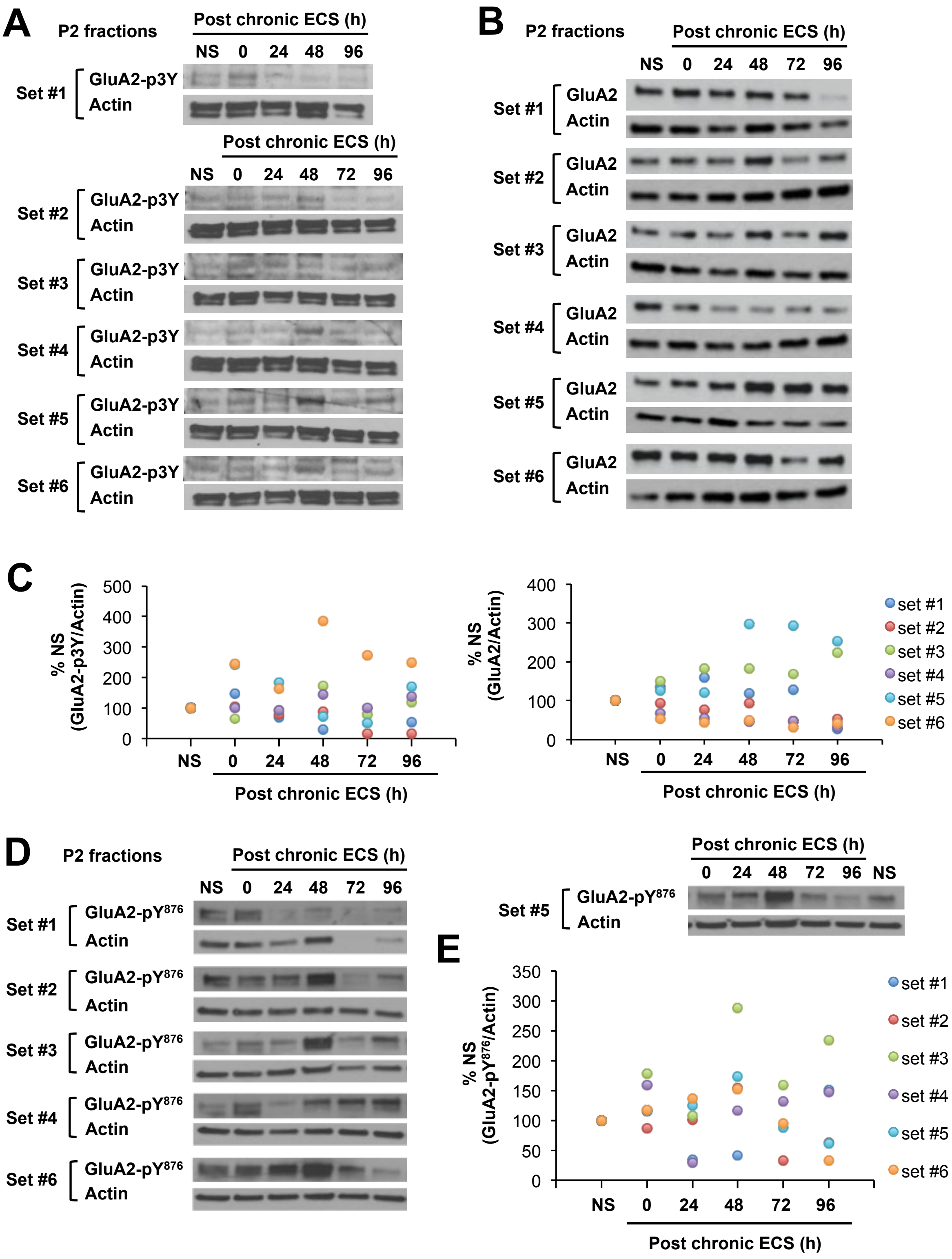

Supplemental Figure 6

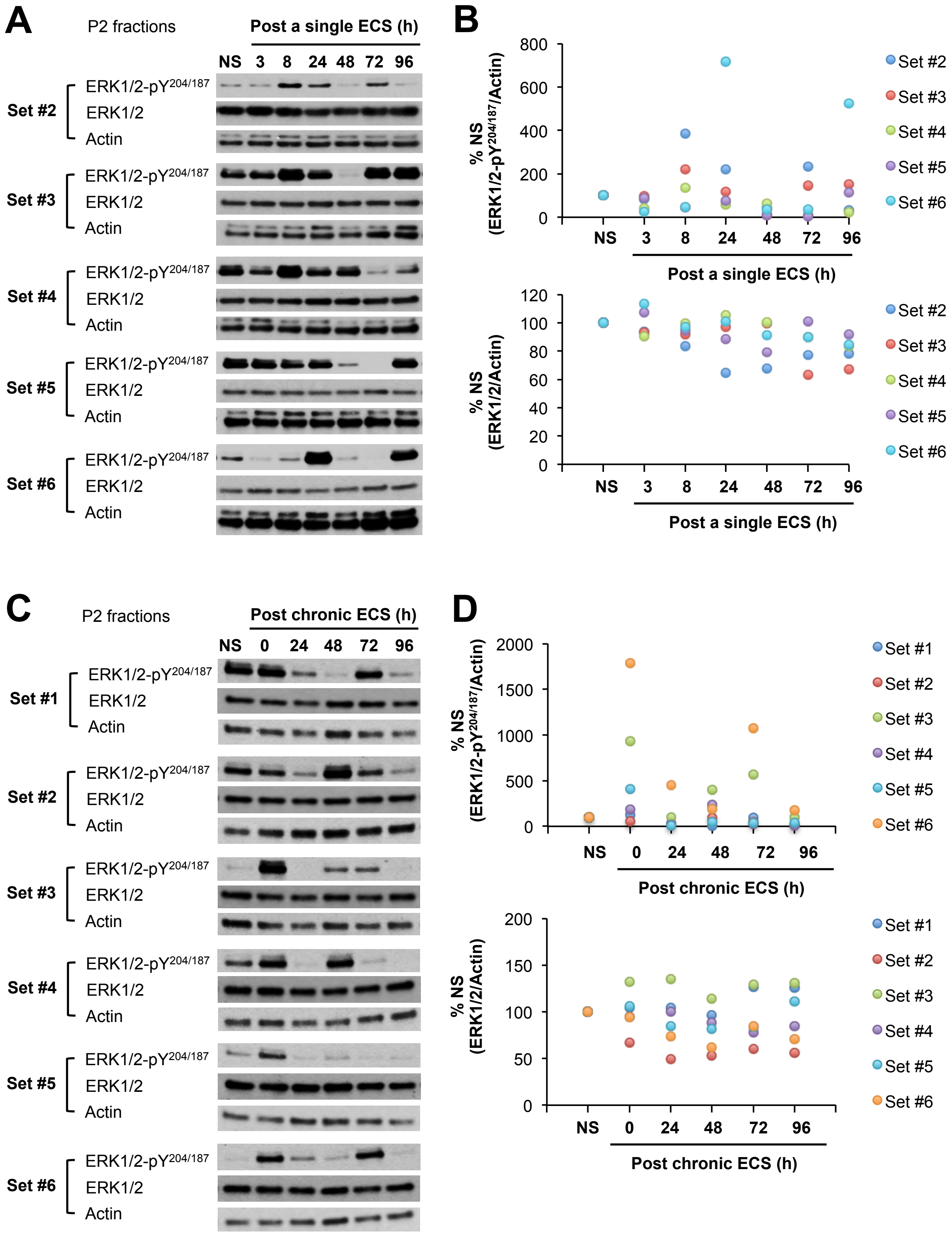

Supplemental Figure 7

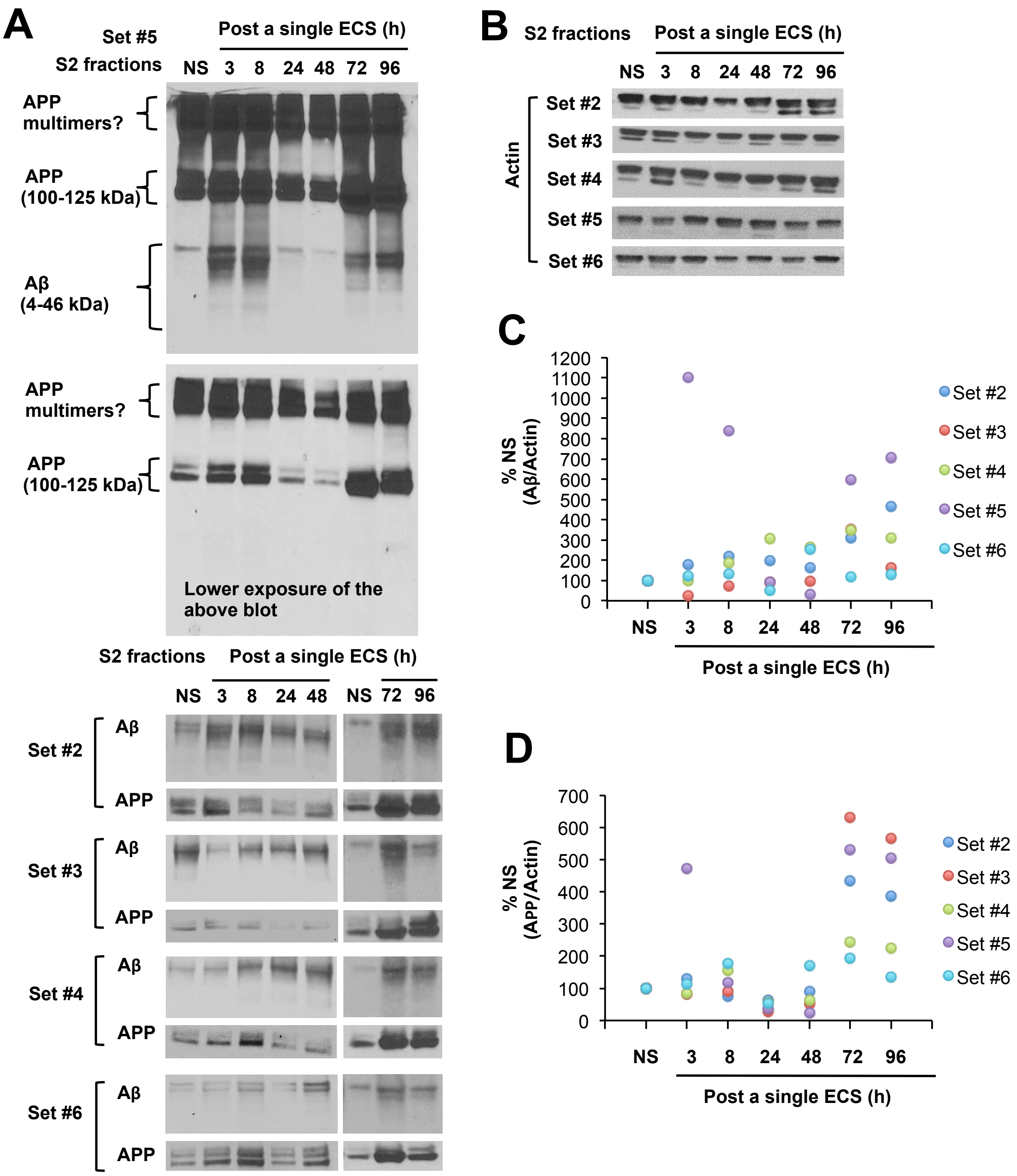

Supplemental Figure 8

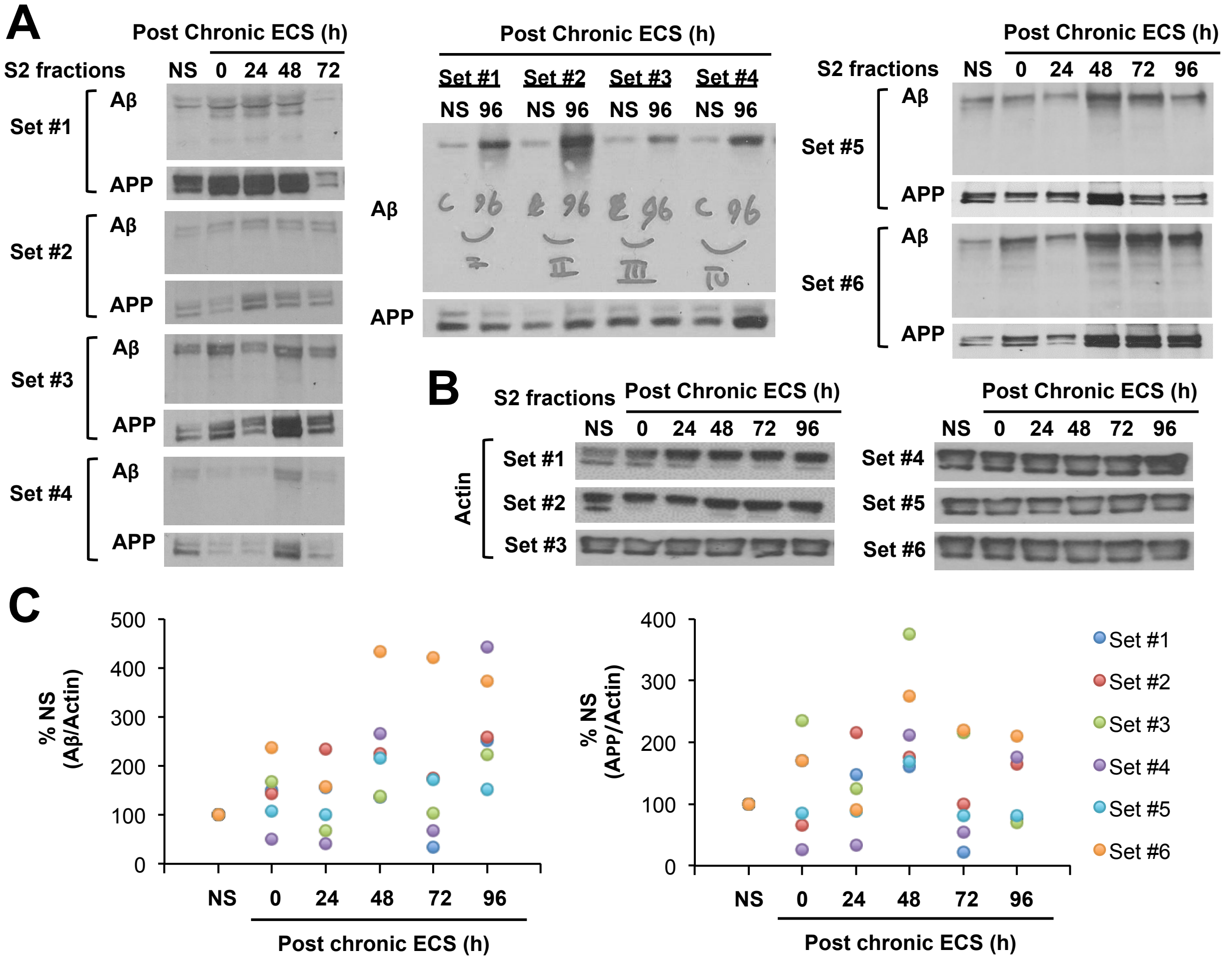

Supplement: Supplementary file 1 — The Supplementary Material shows all raw data collected for Figures 1-5 in the hippocampal membrane P2 fractions of male rats treated with a single ECS and chronic ECS. [file 2123748.f1.pdf]
